# Effect of High Hydrostatic Pressure Processing on the Microbiological Quality and Bacterial Diversity of Sous-Vide-Cooked Cod

**DOI:** 10.3390/foods12061206

**Published:** 2023-03-12

**Authors:** Diego Pérez Alcalá, María José Grande Burgos, Javier Rodríguez López, Rosario Lucas, Antonio Gálvez, Rubén Pérez Pulido

**Affiliations:** Microbiology Division, Department of Health Sciences, Faculty of Experimental Sciences, University of Jaén, 23071 Jaén, Spain

**Keywords:** sous-vide cooking, cod, high hydrostatic pressure, bacterial diversity, microbiological quality, refrigerated storage

## Abstract

High hydrostatic pressure (HP) is a promising method to improve the microbiological quality of sous-vide foods. Monitoring the composition and behavior of the microbial communities in foods is of most importance for the production of high-quality and safe products. High-throughput sequencing (HTS) provides advanced approaches to determine food’s microbial community composition and structure. The aim of the present study was to determine the impact of different HP treatments on the microbial load and bacterial diversity of sous-vide Atlantic cod. Sous-vide cooking at 57.1 °C for 30 min followed by HP treatment at 500 MPa for 8 min reduced viable cell counts (total aerobic mesophiles) in the cod samples below detectable levels for 45 days of storage under refrigeration. In a second trial with cod cooked sous-vide at 52 °C for 20 min followed by HP treatments at 300 or 600 MPa (with HP treatment temperatures of 22 °C or 50 °C for 4 or 8 min, depending on treatment), only the treatments at 600 MPa delayed bacterial growth for at least 30 days under refrigeration. The optimal HP conditions to improve the microbiological quality of sous-vide cod cooked at low temperatures were obtained at 600 MPa for 4 min at a pressurization temperature of 50 °C. Bacterial diversity was studied in cod cooked sous-vide at 52 °C for 20 min by HTS. In the absence of HP treatment, *Proteobacteria* was the main bacterial group. A succession of *Pseudomonadaceae* (*Pseudomonas*) and *Enterobacteriaceae* was observed during storage. *Firmicutes* had low relative abundances and were represented mainly by *Anoxybacillus* (early storage) and *Carnobacterium* (late storage). The HP-treated sous-vide cod showed the greatest differences from controls during late storage, with *Aerococcus* and *Enterococcus* as predominant groups (depending on the HP conditions). The application of HTS provided new insights on the diversity and dynamics of the bacterial communities of sous-vide cod, revealing the presence of bacterial genera not previously described in this food, such as *Anoxybacillus*. The significance of *Anoxybacillus* as a contaminant of seafoods should be further investigated.

## 1. Introduction

Sous-vide cooking is a process where raw foods or half-cooked foods are placed in a plastic pouch or bag, hermetically sealed—generally under a vacuum—and cooked slowly in a water bath under strict temperature control conditions [1]. This method allows better control of changes due to the absence of oxygen and high temperature and improves the shelf-life and the quality of the product, providing a way to satisfy the demands of consumers for “fresh-like” and good-quality processed foods [1]. Since temperature is a critical parameter to maintain the desired textural properties of food, the method does not ensure complete inactivation of foodborne pathogens and spoilage bacteria, and relies to a great extent on the microbiological quality of the raw materials and ingredients and on keeping good hygienic practices during manufacturing.

Several studies have addressed the application of sous-vide cooking to fish and other seafood [1,2,3], such as with fish flakes, where the collagen separating the flakes is converted into gelatin at around 46–49 °C [4]. For that reason, fish is usually cooked at low temperatures. Salmon loins can be cooked at a maximal core temperature around 40 °C in order to assure optimal texture and flavors [5]. Fin and shellfish are often cooked to medium-rare at 49 °C for 15–20 min, while salmon and artic charr may be cooked to rare at 42 °C [6]. These low cooking temperatures do not achieve microbial inactivation, which implies immediate consumption to avoid potential microbiological growth [5]. In order to improve the safety and shelf life of sous-vide products, several hurdles have been proposed such as chill storage, freezing, the addition of natural preservatives such as organic acids, fruit juices or herbal extracts, lactic acid bacteria, bacteriocins, lysozymes, gas packing, irradiation, and the application of high hydrostatic pressure (HP) [2].

High hydrostatic pressure is a non-thermal food processing method for the inactivation of microorganisms that has a low impact on the nutritional value and the organoleptic properties of foods [7]. HP treatments are applied for the reduction of microbial load and shelf-life extension of many different types of ready-to-eat foods, including seafood [8,9,10]. Since HP treatments may affect the properties of protein-rich foods, HP treatments of low intensity (200–350 MPa) are usually employed to minimize the impact on protein texture and color in seafoods. Several studies have reported the effects of HP treatments in the range of 200 to 800 MPa on cod [11,12,13]. Rode and Hovda [14] reported that cod samples treated at 500 MPa presented a total aerobic count undetectable or below two log cycles after 11 days of storage, and the fish still maintained levels below seven log cycles after 26 days. Examples of HP-processed fish products in the market are desalted cod (marketed in Italy in the year 2004 and in Spain in the year 2019) and prepared cod meals (marketed in Spain in 2009) [9]. There is a need to further explore the impact of HP processing on bacterial variations and on the dynamics of bacterial communities in foods. HP also has a great potential to improve the microbiological quality and extend the shelf life of sous-vide food in the food service environment.

There are only a few studies combining sous-vide cooking with HP processing of seafood, such as those carried out on lobster and lobster tails [15,16], whiteleg shrimp [17], salmon loins [5], seabream [18], and largemouth bass [19], and none of them addressed the impact of treatments on the bacterial diversity of the processed food. The application of culture-independent methods such as high-throughput sequencing (HTS) in food microbiological analysis provides much more rich information on the bacterial composition and dynamics compared with culture-dependent methods. To the best of our knowledge, there are no previous studies using HTS on sous-vide seafood processed by HP. Atlantic cod (*Gadus morhua* L.) has been one of the most important commercially fished species for more than a century, and sous-vide cod is a highly appreciated dish. The aim of the present study was to determine the effects of HP treatments on the microbial load of sous-vide cod and to determine the changes in bacterial diversity of cod cooked sous-vide at low temperatures (processed or not by HP) during prolonged storage under refrigeration by using HTS.

## 2. Materials and Methods

### 2.1. Sample Preparation and Treatments

Whole cod fish specimens (Atlantic cod; *Gadus morhua*) of ca. 5.6–5.8 kg were bought the same day of food preparation from the fish market without previous handling, except evisceration. The fish were scaled exhaustively and then washed thoroughly with tap water. The skin and bones were hygienically removed from the loins, and these were cut up aseptically in portions of 25 g each. Such portions were cooled to optimal packaging temperature (Tp) (0 °C < Tp < 3 °C). The pil-pil sauce was prepared as follows: the skin and head of the cod, a few garlic cloves, and some chilies were cooked into extra virgin olive oil (Picual variety) for 30 min at 72 °C. The cooked mix was then sifted and emulsified with a mesh strainer. The resulting pil-pil sauce was cooled to optimal packaging temperature (0 °C < Tp < 3 °C). The loins were lightly seasoned and placed in pil-pil sauce as covering liquid and packed into vacuum sealer bags (polypropylene and polyamide; certified for food use) at a ratio of 25 g of raw cod loin and 8 g of emulsified pil-pil sauce (both at Tp temperature) per bag. Finally, a vacuum pressure of −0.95 bar was applied to the bags and they were heat sealed. Samples prepared as described above were used in two trials including different combinations of heat and high hydrostatic pressure (HP).

In trial 1, all the samples were heated for 30 min in a Garhe Sous Vide model SV-Pro vacuum-cooking system (Garhe S.A., Amorebieta, Vizcaya, Spain) set at a heating temperature of 57.1 °C. The samples were then blast chilled to storage temperature (Ts) in less than 30 min (0 °C < Ts < 3 °C in t < 30′). Half of the samples were stored under refrigeration (4 °C) without further processing, while the rest were processed by HP. Samples were HP-treated in an SFP high pressure system at 500 MPa for 8 min (22 °C). After an almost-immediate decompression, the samples were chilled on ice and then stored under refrigeration. Non-pressurized and pressurized batches were sampled for analysis for 60 days.

Since several chefs prefer cooking fish at lower temperatures, a second trial (trial 2) was carried out in which the samples were heated at 52 °C for 20 min followed by blast chilling as described above. In total, 20% of the samples were stored under refrigeration without further processing, while the rest were used for high hydrostatic pressure (HP) processing. The following HP treatments were applied in trial 2: A, 300 MPa for 8 min at 50 °C; B, 600 MPa for 4 min at 50 °C; C, 600 MPa for 8 min at 22 °C; D, 600 MPa for 8 min at 50 °C. Right after application of treatments, the samples were chilled on ice before they were stored under refrigeration. All samples were stored under refrigeration for up to 105 days and sampled for analysis (first at days 3, 7, 14, and 30, and then periodically every 15 days), as will be described below.

### 2.2. Determination of Viable Cell Concentrations

Controls and HP-treated samples, in duplicate, were removed from cold storage and mixed by hand while they were still in the vacuum-package bag. Then, 25 g of the obtained mixture of fish tissue and sauce was transferred to a stomacher bag and homogenized with 25 mL of buffered peptone water (BPW) for 1 min. The homogenate was serially diluted with sterile saline solution (SS) and plated in triplicate on culture media as follows. In trial 1, plating was conducted on trypticase soya agar (TSA, Scharlab, Barcelona, Spain) for total aerobic mesophiles, saline TSA (prepared by supplementing TSA with 10 g/L NaCl), and MacConkey agar (Scharlab) for presumptive *Enterobacteriaceae*. The following media were used for viable counts in trial 2 (based on results obtained in trial 1): TSA and MacConkey agar. The plates were incubated at 37 °C for 24–48 h. The average number of colonies was used to calculate the concentration of viable cells in the sample (as log_10_ colony-forming units, CFU per gram of sample).

### 2.3. DNA Isolation and Quantification

Sample homogenates corresponding to trial 2 were used for DNA extraction and study of bacterial diversity. An aliquot (5 mL) of the sample homogenates obtained in Section 2.2 was mixed with 45 mL sterile saline solution in MilliQ-grade water and centrifuged at 500× *g* for 5 min in a Beckman GS-6R centrifuge to remove food debris. The supernatant was collected and centrifuged at 6000× *g* for 30 min to recover microbial cells. The pellets were resuspended in 1 mL sterile saline solution and centrifuged at 500× *g* for 1 min in an Eppendorf centrifuge 5424R. The supernatants were transferred to a clean Eppendorf tube and centrifuged at 13,500× *g* for 5 min. The resulting pellets were resuspended in 0.5 mL sterile saline solution and treated with propidium monoazide (PMA™, GenIUL, S.L, Barcelona, Spain) [20,21]. After PMA treatment, the samples were washed twice with sterile saline solution and once with MilliQ-grade water (by centrifugation at 13,500× *g* for 5 min). The samples were stored at −20 °C and then total DNA was extracted by using a GenElute™ DNA extraction kit (Sigma-Aldrich, Burlington, MA, USA) following the manufacturer’s instructions. QuantiFluor^®^ ONE dsDNA system (Promega, Madison, WI, USA) was used to determine the DNA concentration and quality. The DNA corresponding to the two biological replicates (same treatment and storage time) was pooled into a single sample for DNA sequencing and determination of bacterial diversity as described below.

### 2.4. DNA Sequencing and Analysis

The 16S-rRNA gene V3-V4 hypervariable regions were amplified with modified primers 341F and 805R [22] to analyze the bacterial communities in the samples by HTS. The procedures for library preparation, quality assessment and sequence joining, bioinformatic analysis, and metataxonomic analysis were described in previous work [23]. The libraries were sequenced using a 2 × 300 bp paired-end run (MiSeq Reagent kit v3 (MS-102-3001)) on a MiSeq Sequencer according to the manufacturer’s instructions (Illumina, Inc., San Diego, CA, USA). The database used for this taxonomic assignation was the SILVA_release_132. The abundances of operational taxonomic units (OTUs) were estimated after the classification.

### 2.5. Statistical Analysis

Data were analyzed with SPSS software v. 24 (IBM Corp., Foster City, CA, USA) and Excel program (Microsoft Excel 2019, Microsoft Corporation, Redmond, WA, USA). The statistical significance (*n* = 6; *p* < 0.05) was determined.

## 3. Results

### 3.1. Effect of Treatments on Microbial Loads

Control samples from trial one showed viable counts of total aerobic mesophiles that were below or close to the detection limit for the first 14 days of storage (Table 1). After that point, counts increased significantly (*p* < 0.05) with storage, reaching values above 5 log CFU/g by day 45. In the pressurized samples, viable count remained below the detection limit for the first 45 days of storage and approached 5 log CFU/g by day 60. Viable counts obtained on TSA did not differ significantly (*p* > 0.05) from counts obtained on saline TSA for any of the samples. Viable cell counts on MacConkey agar were below the detection limit for the whole storage period in all the samples (including controls and pressurized samples).

The results of viable cell counts obtained for samples corresponding to trial two are shown in Table 2. Since saline TSA did not provide significant differences in viable counts compared with TSA according to results from trial one, we only used TSA in trial two. The microbial load of the control samples from trial two increased significantly during storage, reaching 5.5 log CFU/g by day 7 and 6.5 log CFU/g by day 14 (Table 2).

During prolonged incubation for up to 105 days, counts corresponding to trial two reached a plateau of 10.5 to 10.9 log CFU/g (days 60 to 90). Counts obtained on MacConkey agar (presumptive *Enterobacteriaceae*) also increased significantly during storage, reaching 4.7 log CFU/g by day 14 and up to 9.7 log CFU/g at day 75. The pressurized samples only yielded viable counts on TSA, but not on MacConkey agar. Right after treatments, counts on TSA were below the detection limit for all treatment conditions (Table 2). At day three of storage, viable counts were very low and close to the limits of detection, ranging from 1.6 to 2.1 log CFU/g for the treatment of highest intensity (600 MPA, 8 min, 50 °C) and the lowest (300 MPA, 8 min, 50 °C), respectively. After that, there was an incubation period (days 7 to 30) in which total viable counts were below the detection limit for all treatments. From day 45 on, growth was detected in all the treated samples. However, significant differences in viable counts were always detected between treatments (Table 2). During the remaining incubation period, viable counts were highest for the lowest intensity treatment (300 MPa, 8 min at 50 °C) followed by treatment at 600 MPa for 8 min at 22 °C. The treatments at 600 MPa and 50 °C were the most effective, especially the treatment of longer duration (600 MPa for 8 min at 50 °C). For this last treatment, viable counts were still below 5 log CFU/g at day 60 of refrigerated storage, and reached a maximum of 5.3 log CFU/g at day 75. The treatment at 600 MPa for 4 min at 50 °C was also quite effective at delaying bacterial recovery, with counts reaching 5.7 log CFU/g at day 75. By contrast, for the less effective treatment counts reached values from 8.5 (day 75) to 8.8 (day 90) log CFU/g.

### 3.2. Changes in Bacterial Diversity

The bacterial diversity of samples corresponding to trial two was studied. The number of reads assigned to operational taxonomic units (OTUs) at the genus level ranged from 37,162 to 129,557, with an average of 76,881. For the control samples, the Chao1 index decreased from the seventh day of incubation on. For the rest of the samples, decreases in the Chao1 index were observed after longer incubation times (usually after days 30, 45, or 60, depending on treatment). Decreases in Shannon and Simpson indexes were also observed during late incubation (Supplementary Table S1).

**Figure 1:**
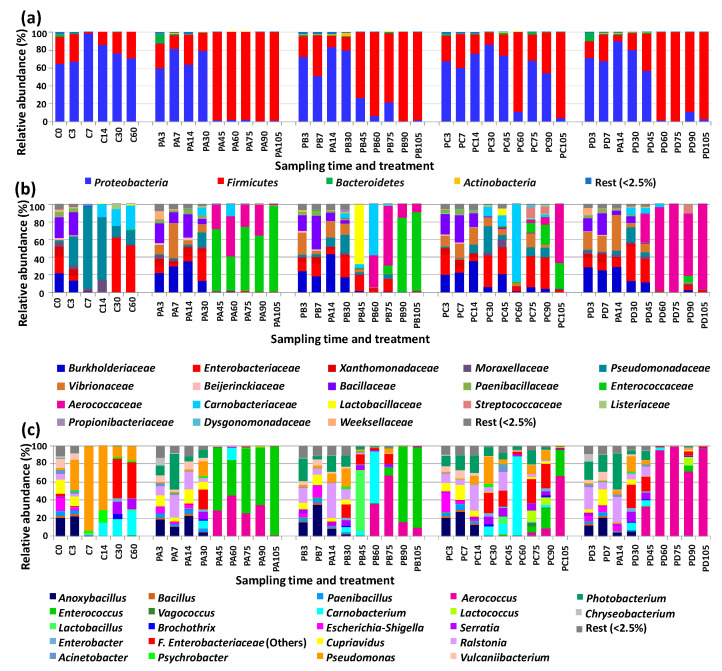
Relative abundances at the phylum (**a**), family (**b**), and genus (**c**) level of the main bacterial groups of cod samples corresponding to trial 2 at different storage times. C, controls. Treatments: PA (300 MPa, 8 min, 50 °C); PB (600 MPa, 4 min, 50 °C); PC (600 MPa, 8 min, 22 °C); PD (600 MPa, 8 min, 50 °C).

The microbiota of control samples was defined mainly by *Proteobacteria*, together with *Firmicutes*. *Bacteroidetes* and *Actinobacteria* had low abundances (Figure 1a). The relative abundances of these phyla as well as the lower taxa changed during the refrigerated storage period. At storage time zero, *Proteobacteria* included the following main families (by order of relative abundance): *Burkholderiaceae* (represented by the genera *Cupriavidus* and *Ralstonia*), *Enterobacteriaceae* (represented by *Escherichia-Shigella* and others—assigned only to the family level), *Xanthomonadaceae* (gen. *Vulcaniibacterium*), and *Moraxellaceae* (gen. *Acinetobacter*) (Figure 1b,c). *Firmicutes* were represented mainly by the families *Bacillaceae* (gen. *Anoxybacillus*) and *Paenibacillaceae* (gen. *Paenibacillus*). During storage of the control samples, *Proteobacteria* had the highest relative abundances at days 7 and 14. In this early storage period, *Pseudomonadaceae* (gen. *Pseudomonas*) increased in relative abundance from day three and became the predominant group at days seven and fourteen. Members of *Burkholderiaceae* still had high relative abundances at day three, but then decreased drastically in samples corresponding to storage days 7 to 60. A similar change was observed for *Xanthomonadaceae*. *Enterobacteriaceae* decreased markedly at days 7 and 14, but then became the predominant group during the remaining storage period sampled (with relative abundances above 50%). During this late incubation period, the main representatives were *Enterobacteriaceae* (others) followed by *Enterobacter* and *Serratia*. Regarding *Firmicutes*, endospore formers still had high relative abundances at day three, but then decreased markedly for the following storage days. Remarkably, members of Fam. *Carnobacteriaceae* (gen. *Carnobacterium*) became more abundant from day 14 on, reaching 27.5% at day 60.

The microbiota of the pressurized samples were mainly composed of members of *Proteobacteria* for the first 30 days of storage (Figure 1a). After that, *Fimicutes* became the predominant group, with some exceptions (treatment C at days 45, 75, and 90 and treatment D at day 45). Fam. *Burkholderiaceae* (genera *Cupriavidus* and *Ralstonia*) showed the highest relative abundances in the pressurized samples for the first 14 days of storage (Figure 1b,c). *Vibrionaceae* (gen. *Photobacterium*) also had high relative abundances during this early storage period, while *Enterobacteriaceae* had the highest relative abundances at day 30 or at days 30 and 45 (depending on treatment). *Pseudomonadaceae* (gen. *Pseudomonas*) had low relative abundances in most of the pressurized samples, except at day 30 of storage.

*Firmicutes* were represented in the pressurized samples mainly of members of fam. *Bacillaceae* (gen. *Anoxybacillus*) during early storage (up to day 14). During mid to late storage, the main representatives of *Firmicutes* were *Aerococcaceae* (gen. *Aerococcus*), *Enterococcaceae* (gen. *Enterococcus*), and *Carnobacteriaceae* (gen. *Carnobacterium*). The relative abundances of these three main bacterial groups changed considerably depending on the pressure treatment applied. *Enterococcus* had the highest relative abundances in the samples treated at lower pressure (300 MPa, 8 min, 50 °C) and at two storage points of the samples treated at 600 MPa for 4 min (50 °C). *Aerococcus* was the predominant genus detected during late storage (days 60 to 105) in the samples with the highest intensity treatment (600 MPa, 8 min, 50 °C). It was also the most abundant genus in two samples from other treatments at 600 MPa (PB75 and PC105). *Carnobacterium* also had the highest relative abundance in two samples pressurized at 600 MPa (PB60 and PC60).

**Figure 2:**
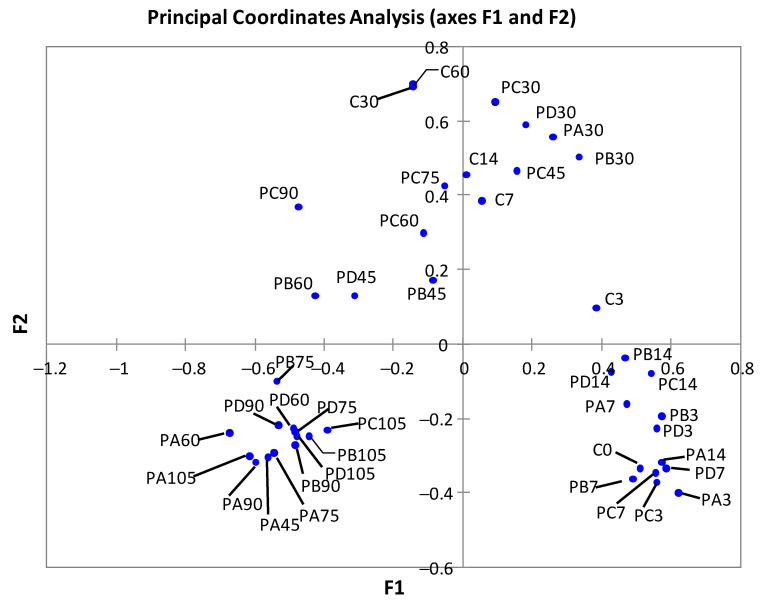
Results obtained after principal coordinates analysis of cod samples from trial 2 according to bacterial diversity data at genus level at different days of refrigerated storage. C, controls. Treatments: PA (300 MPa, 8 min, 50 °C); PB (600 MPa, 4 min, 50 °C); PC (600 MPa, 8 min, 22 °C); PD (600 MPa, 8 min, 50 °C).

The principal coordinates analysis showed a progressive separation of control samples as incubation time increased (Figure 2). The last two samples (times 30 and 60) were very close, in agreement with their similar microbial composition. The pressurized samples corresponding to the early period of storage (days 3 to 14) mapped closely and also close to the control samples at time zero. The pressurized samples stored for 30 days also mapped in a separate group. Most of the pressurized samples from late storage also mapped closely, especially the PA and PD samples.

## 4. Discussion

Results from the present study indicated that the microbiological quality of sous-vide cod was markedly influenced by the initial heating process and the parameters of the high hydrostatic pressure (HP) applied. Remarkably, all HP treatments tested reduced *Enterobacteriaceae* below detectable levels during storage, an indication of the improvement in the safety of the food. HP treatment at 500 MPa for 8 min of cod cooked sous-vide at 57.1 °C for 30 min provided the additional benefit of keeping the microbial counts of total aerobic mesophiles below detectable levels for at least 45 days compared with the non-pressurized samples. This combination could be satisfactory to extend the product’s shelf life. Nevertheless, there is work in the literature indicating that some chefs prefer cooking fish at low temperatures between 42 and 49 °C that do not achieve microbial inactivation [6]. Since there is a tendency to decrease the cooking temperature of sous-vide foods (which may indeed require longer cooking times), we tested low-temperature short-time cooking (52 °C for 20 min) in combination with different HP conditions (including, at the same time, the combination of HP with moderate heat in order to increase microbial inactivation). In this case, the lower intensity of the sous-vide cooking left a higher surviving population; therefore, bacterial growth in the non-pressurized samples was much faster. Because of this higher microbial load, the HP treatments were less effective, and only the treatments of highest intensities (B, C, D) were able to maintain the surviving fraction in the pressurized samples below 5 log CFU/g for at least 45 days. These results outline the importance of sous-vide cooking temperature for the microbiological quality of the product, even if an HP treatment is going to be applied afterwards. Reducing the microbial load of food products is essential to maintain the food quality and safety. According to FDA guidelines, aerobic plate counts of 5 log CFU/g are still acceptable for fresh-, frozen-, and cold-smoked fish [24]. However, European Commission regulations [25,26] focus on histamine levels and not as much on the total microbial load of fish.

Previous studies have reported on the beneficial effects of the application of HP treatments to sous-vide cooked fish. Espinosa et al. [18] reported that HP treatment at 600 MPa for 5 min at 5 °C enhanced the texture (firmness), and thus improved the sensory quality of seabream cooked sous-vide at 65 °C. Picouet et al. [5] sous-vide cooked fresh salmon loins until the core temperature reached 40 °C, followed by cooling and application of HP treatments at 210, 310, and 400 MPa for 300 s at 10 °C. The authors found that pressure above 310 MPa could reduce the initial microbial load and prolong the shelf life to 6 days. HP treatments of sous-vide cooked salmon loins at 210, 310, and 400 MPa resulted in a 0.8, 1.0, and 2.2 log CFU/g reduction in the total viable count. Zhou et al. [19] reported that the application of HP treatments at 400 MPa for 0 and 10 min at 25 °C on largemouth bass cooked sous-vide at 90 °C for 15 min effectively inhibited microbial growth for 30 days at 4 °C.

The previous studies carried out on sous-vide fish were based on the application of HP treatments either at temperatures close to ambient conditions or at low temperatures (5 °C or 10 °C). However, the efficacy of HP can be improved by increasing the treatment temperature. This is because temperatures close to 50 °C or higher destabilize the bacterial membranes, making the bacterial cells more sensitive to HP. Thus, HP is often combined with other hurdles (such as mild heat treatment) to keep the pressure moderate not only to reduce the undesirable effects of high pressure on food quality [27], but also to improve microbial inactivation. Results from the present study indicate that increasing the temperature of HP treatments to 50 °C caused greater cell damage and reduced microbial proliferation in the sous-vide cod during storage compared with treatment carried out at 22 °C. Since most industrial HP systems can operate at temperatures up to 80–90 °C, the combination of sous-vide cooking at low temperatures with HP-heat could be an interesting approach to prolong the shelf life of sous-vide fish for restoration and catering services. The combination of HP with sous-vide cooking could also add benefits as a barrier against foodborne pathogens that are able to grow during refrigerated storage, mainly *Listeria monocytogenes* and non-proteolytic *Clostridium botulinum*. Although bacterial endospores are pressure-resistant, the sous-vide cooking step may activate endospore germination. During germination, resistance to environmental factors (such as antimicrobials, heat, radiation, and HP) decreases, making the bacterium sensitive to HP treatments. Furthermore, studies have reported a lower pressure resistance of bacterial endospores when HP is used in combination with heat [28,29,30]. Lenz et al. [29] reported reductions in viable counts for *C. botulinum* type E endospores suspended in imidazole phosphate buffer (pH 7) of ca. 1 log unit for 600 s HP treatments at 450 MPa and 45 °C or ca. 1.5 log units for 600 MPa at 60 °C. The authors also reported that endospore inactivation increased greatly as the pressure and temperature of treatments increased. These results were similar to those reported in low-acid foods [30]. Since *C. botulinum* (specially the non-proteolytic strains) can be a matter of concern in sous-vide-cooked cod during refrigerated storage, further experiments need to be carried out, including challenge tests with *C. botulinum* endospores in order to determine the effects of sous-vide cooking followed by HP treatments on endospore inactivation. Yet, data obtained from culture-independent analyses indicated that reads assigned to gen. *Clostridium* sensu stricto were only detected in four of the samples, and they had very low relative abundances of ≤0.1%.

In order to gain more information on the microbial composition of the sous-vide-cooked samples, we chose to apply high-throughput sequencing (HTS) technology on samples from trial two. Trial two was selected because we could detect survivors on both the controls and the pressurized samples, which allows a better comparison and elucidation of the effect of HP treatments on bacterial populations. The results obtained by the culture-independent analysis indicated that the mild heat treatment applied to the control cod samples (52 °C for 20 min as the only treatment) left a complex population in which the three main bacterial groups detected were the Gammaproteobacteria families *Burkholderiaceae* and *Enterobacteriaceae*, and the fam. *Bacillaceae*.

The main representative of fam. *Bacillaceae* was *Anoxybacillus*. This genus was found not only in the control samples during early storage (times zero and three), but also in the pressurized samples (extending up to day 30 of storage). *Anoxybacillus* has mostly been isolated from hot springs, where it may be the dominant genus [31]. The majority of the sequence reads obtained in the present study for this genus were assigned to the species *Anoxybacillus flavithermus* subsp. *flavithermus*. Given the thermophilic trait of this bacterium, we can suspect that cod contamination with *A. flavithermus* may be a contaminant from the food cooking environment. The ability of the bacterium to grow at moderate temperatures (optimum range 50–65 °C) and to elicit strategies (biofilm formation, production of endospores) to survive disinfection and/or heat makes us suspect that it could also colonize kitchens and cooking utensils (similarly to what happens in the dairy industry). A recent study correlated *Anoxybacillus* with gene expression in the gut of gilthead sea bream [32]. Tentatively, the fish gut microbiota could be the source of contamination with *Anoxybacillus* for the fish and the fish-processing environment. These results are encouraging for further studies on the incidence of *Anoxybacillus* in cod and other types of fish and its possible role in the spoilage of seafood products.

Among *Enterobacteriaceae*, most of the OTUs for days zero and three were assigned to *Escherichia-Shigella*, which is worrying since these bacteria can produce foodborne illness. This group was also found in the pressurized samples with high relative abundances (except in the samples treated at 600 MPa for 8 min at 50 °C) for the first 7 days of storage. Previous studies have reported on the pressure-resistance of *E. coli* [33,34], which could be an explanation for the results obtained on the pressurized samples. However, *Enterobacteriaceae* were not detected in any of the pressurized samples by the culture-dependent analyses, suggesting that they could be sublethally injured and unable to grow in the selective medium. The absence of detectable *Enterobacteriaceae* during the whole storage period would also suggest that the sublethally injured cells were unable to repair cell damage in the refrigerated cod.

The initial microbiota found in the control samples was replaced by other bacterial groups during early storage of the samples (days three to seven) as the product became spoiled. Remarkably, *Pseudomonas* was the main spoiling bacterium detected in the control samples from storage times 3 to 14, with the highest relative abundance at day seven. Contamination with *Pseudomonas* may have occurred from the fish processing environment. *Pseudomonas* has been isolated from many different seafoods and predominates in marine fish stored under refrigerated aerobic conditions where it can rapidly outgrow other spoilage bacteria [35]. In the present study, most of the OTUs from gen. *Pseudomonas* were assigned to *Pseudomonas fragi*, which is among the *Pseudomonas* species commonly detected and associated with seafood spoilage [36].

The microbiota that develops during fish spoilage depends greatly on the storage conditions and duration of storage. Previous studies have reported that *Pseudomonas* spp. and *Psychrobacter* spp. dominated the spoilage microbiota of chilled cod in air, while *Carnobacterium maltaromaticum* and *Rahnella aquatilis* dominated the microbiota of chilled cod packed under modified atmosphere [37]. However, we have found no previous studies on spoilage bacteria from lightly cooked cod. In the present study, OTUs assigned to gen. *Psychrobacter* only had a high relative abundance at day 14, together with *Carnobacterium*. *Carnobacterium* and *Enterobacteriaceae* were the main groups displacing *Pseudomonas* towards the end of storage (days 30 and 60). *Carnobacterium* may cause rapid spoilage of seafood and they can also produce volatile compounds such as of 2,3-butanedione diacetyl (butter smell) and 2,3-pentanedione [38].

The main difference in the microbial dynamics of the pressurized samples compared with control samples was that the major spoilage groups detected in control samples (that is, *Pseudomonas* and *Enterobacteriaceae*) had very low relative abundances in the pressurized samples. As mentioned above, the microbiota of the pressurized samples during early storage included many of the bacterial groups also detected in the control samples (such as *Escherichia-Shigella* and *Anoxybacillus*). However, the microbiota of the pressurized cod samples during late storage were composed mainly of lactic acid bacteria (LAB) of the families *Aerococcaceae* and *Enterococcaceae* and, at some points, also of *Carnobacteriaceae, Lactobacillaceae*, and *Streptococcaceae*. The relative abundance of *Lactobacillales* was in the range of 97.7% to 99.3% in the period comprised between days 45 and 105 for treatment A or 76.8 to 99.3 for treatment B. Similarly, *Lactobacillales* reached 87.4 to 99.5% at days 60 to 105, and treatment C showed two peaks of 88.5% at day 60 and 95.8% at day 105. The relative abundances of the main LAB found during late storage depended greatly on the pressure conditions applied. For example, *Enterococcus* was the predominant group in the lowest intensity treatment A, followed by *Aerococcus* for most of the samples (except PA60) compared with only the last two samples of treatment B (PB90, PB105). By contrast, samples from treatment B showed high relative abundances of other LAB (e.g., *Lactobacillus* in sample PB45 and *Carnobacterium* in PB60 or *Aerococcus* in PB60). Sample C was more heterogeneous, with *Carnobacterium* as the main group at PC60 and *Aerococcus* followed by *Enterococcus* at PC105. In the treatment of highest intensity (D), *Aerococcus* was clearly the main bacterial group at the end of storage, while the rest of the lactic acid bacteria had very low relative abundances. There are no previous studies on the pressure resistance of *Aerococcus.* The results obtained could be explained taking into consideration several factors such as the sensitivity of the particular bacteria to the HP treatment (intensity, duration, and temperature of HP treatment), their capacity for proliferation in the refrigerated samples, and competition with other survivors also present in the samples.

The significance of LAB at the end of the storage period of the pressurized cod samples needs to be analyzed. LAB are known as a group of Gram-positive, catalase-negative, nonsporulating, aero-tolerant, acid-tolerant, and strictly fermentative cocci or rods with an array of benefits in foods [39]. For example, they are generally recognized as safe, many of them play an important role in food preservation due to different mechanisms for the displacement of pathogens (such as competition for nutrients, acidification, and production of an array of antimicrobial substances), and some may have health-promoting effects [39]. At the same time, LAB may cause fish spoilage, including fish packed under a vacuum or modified atmosphere, as well as lightly preserved seafood [40,41,42].

The most predominant LAB in fish products are *Lactobacillus*, mainly of the species *L. sakei*, *L. curvatus*, and *Carnobacterium*, mainly *C. maltaromaticum*, although *Leuconostoc* and *Lactococcus* are also found sometimes [41]. In the pressurized samples from the present study, most of the OTUs assigned to gen. *Lactobacillus* belonged to the species *L. sakei*. LAB may cause fish spoilage due to the release of a variety of metabolic products from the metabolism of carbohydrates, organic acids, and amino acids [41]. In addition, some (particularly *Enterococcus*) may produce biogenic amines from the decarboxylation of amino acids, which has special relevance in protein-rich products such as fish. On the other hand, LAB may also produce an array of antimicrobial substances that make them interesting for the biopreservation of seafoods.

Results from the present study also indicated that *Aerococcus* was a predominant LAB in some of the pressurized samples during storage. Yet, *Aerococcus* has not been recognized as a relevant genus in fish products. This bacterium is widely distributed as part of the normal microbiota of healthy freshwater fish and has been isolated from salted cod [43] and smoked salmon [44]. The bacterium was reported by culture-independent methods from shrimp paste fermentation [45]. Some *Aerococcus* species can be pathogenic to crustaceans, and one study documented the role of *A. viridans* in pathogenesis of cultured tilapia [46].

*Aerococcus* species deserve additional interest due to their potential for the biopreservation of fishery products [44]. One study reported that *A. viridans* strain SF1044 from fresh salmon was able to inhibit fish spoilage bacteria and did not induce spoilage off-odor [44]. In light of the results obtained in the present work, where *Aerococcus* was a relevant group during late storage of pressurized cod samples, it would be convenient to carry out culture-dependent studies to determine the potential application of the isolates in food preservation.

## 5. Conclusions

Sous-vide cooking of Atlantic cod at 57.1 °C for 30 min followed by HP treatment at 500 MPa for 8 min is an efficient treatment to keep the microbiological quality of the product at acceptable levels for 45 days of storage under refrigeration. The cooking temperature is a key step for product quality. When the cod is cooked sous-vide at a lower temperature of 52 °C for 20 min, the proliferation of microorganisms during storage is faster and the product shelf life is considerably shorter (of less than 7 days). The application of HP treatments on cod cooked at lower temperatures delays bacterial growth considerably and extends the product’s shelf life for at least 30 days. The best results for sous-vide cooking at low temperatures were obtained in combination with an HP treatment at 600 MPa for 4 min at a pressurization temperature of 50 °C. Therefore, this combination of pressure and temperature could be recommended in order to improve the preservation of cod cooked sous-vide at a low temperature. These results may be of interest for the restaurant and catering industries. Results of this study also indicate that sous-vide-cooked cod can support the growth of complex bacterial communities during refrigerated storage, whose composition and succession greatly depend on the intensity and conditions of the HP treatment being applied. The application of HTS provides information on the changes in bacterial communities of sous-vide cod that cannot be obtained readily by culture-dependent methods. The information obtained by HTS on the bacterial profiles of the food during storage under refrigeration can be important for better understanding food spoilage processes.

## Figures and Tables

**Table 1:** Results for trial 1. Viable cell counts (log CFU/g) obtained on non-selective and on selective media for the controls (cod heated at 57.1 °C for 30 min) and the samples processed by HP.

Time (Days)	Control (TSA)	Control (Saline TSA)	Control (MacConkey)	HP (500 MPa, 8 min; TSA)	HP (500 MPa, 8 min; Saline TSA)	HP (500 MPa, 8 min; MacConkey)
0	<1.30	<1.30	<1.30	<1.30	<1.30	<1.30
3	1.70 ± 1.41 ^a^	1.70 ± 1.41 ^a^	<1.30	<1.30	<1.30	<1.30
7	1.70 ± 1.41 ^a^	1.70 ± 1.41 ^a^	<1.30	<1.30	<1.30	<1.30
14	1.70 ± 1.41 ^a^	2.18 ± 0.21 ^a^	<1.30	<1.30	<1.30	<1.30
30	3.70 ± 0.03 ^a^	3.78 ± 0.15 ^a^	<1.30	<1.30	<1.30	<1.30
45	5.63 ± 0.06 ^a^	5.69 ± 0.02 ^a^	<1.30	<1.30	<1.30	<1.30
60	7.33 ± 0.03 ^a^	7.40 ± 0.01 ^a^	<1.30	4.92 ± 0.03 ^b^	4.98 ± 0.01 ^b^	<1.30

^a^, non-significant difference for the same sampling point. ^b^, significantly lower than controls for the same sampling point.

**Table 2:** Results obtained for trial 2. Viable cell counts (log CFU/g) obtained on non-selective and on selective media for the controls (cod heated at 52 °C for 20 min) and for samples processed by different HP treatments.

Time (Days)	Control (TSA)	Control (MacConkey)	Treatment A	Treatment B	Treatment C	Treatment D
0	2.30 ± 0.29 ^a^	1.90 ± 0.30 ^a^	<1.30	<1.30	<1.30	<1.30
3	3.13 ± 0.22 ^a^	2.86 ± 0.24 ^a^	2.13 ± 0.30 ^b^	1.86 ± 0.45 ^b^	2.02 ± 0.41 ^b^	1.59 ± 0.35 ^b,c,d^
7	5.45 ± 0.06	3.70 ± 0.13 ^b^	<1.30	<1.30	<1.30	<1.30
14	6.45 ± 0.06	4.70 ± 0.14 ^b^	<1.30	<1.30	<1.30	<1.30
30	8.73 ± 0.03	7.71 ± 0.05 ^b^	<1.30	<1.30	<1.30	<1.30
45	9.59 ± 0.15 ^a^	9.21 ± 0.43 ^a^	4.99 ± 0.13 ^b^	2.38 ± 0.14 ^b,c,d^	3.04 ± 0.09 ^b,c^	1.85 ± 0.39 ^b,c,d,e^
60	10.51 ± 0.44	9.68 ± 0.15 ^b^	6.70 ± 0.04 ^b^	4.79 ± 0.06 ^b,c^	5.17 ± 0.05 ^b,c^	4.26 ± 0.10 ^b,c,d,e^
75	11.03 ± 0.38	9.56 ± 0.07 ^b^	8.47 ± 0.10 ^b^	5.67 ± 0.06 ^b,c,d^	6.54 ± 0.05 ^b,c^	5.37 ± 0.07 ^b,c,d^
90	11.64 ± 0.09	10.45 ± 0.39 ^b^	8.77 ± 0.11 ^b^	5.67 ± 0.07 ^b,c,d^	7.07 ± 0.14 ^b,c^	5.10 ± 0.17 ^b,c,d,e^
105	9.79 ± 0.48	8.84 ± 0.10 ^b^	6.55 ± 0.05 ^b^	5.27 ± 0.15 ^b,c^	5.64 ± 0.23 ^b,c^	4.58 ± 0.20 ^b,c,d,e^

Treatments: A (300 MPa, 8 min, 50 °C); B (600 MPa, 4 min, 50 °C); C (600 MPa, 8 min, 22 °C); D (600 MPa, 8 min, 50 °C). ^a^, non-significant difference for the same sampling point. ^b^, significantly different than controls for the same sampling point. ^c^, significantly lower than samples treated at 300 MPa, 8 min, 50 °C for the same sampling point. ^d^, significantly lower than samples treated at 600 MPa, 8 min, 22 °C for the same sampling point. ^e^, significantly lower than samples treated at 600 MPa, 4 min, 50 °C for the same sampling point.

## Data Availability

The data presented in this study are available on request from the corresponding author. The data are not publicly available for proprietary reasons.

## Supplementary Materials

The following supporting information can be downloaded at: https://www.mdpi.com/article/10.3390/foods12061206/s1, Table S1: Number of assigned reads and alpha diversity indexes determined at genus level of controls (C) and pressurized (P) samples.
