# Supplementary material for: Effect of High Hydrostatic Pressure Processing on the Microbiological Quality and Bacterial Diversity of Sous-Vide-Cooked Cod
Source: Foods. 2023 Mar 12;12(6):1206. doi: 10.3390/foods12061206 (PMC10047930; doi:10.3390/foods12061206)
Supplement: Supplementary file 1 [file foods-12-01206-s001.zip › foods-2220867-supplementary.pdf]

Supplementary Table S1. Number of assigned reads and alpha diversity indexes determined at genus level of controls (C) and pressurized (P) samples. PA (300 MPa, 8 min., 50 °C); PB (600 MPa, 4 min., 50 °C); PC (600 MPa, 8 min., 22 °C); PD (600 MPa, 8 min., 50 °C).

Sample	Nº reads	CHAO1	Shannon	Simpson
C0	57650	69	2.67	0.89
C3	83152	67	2.32	0.83
C7	105751	33	0.36	0.12
C14	91869	14	0.82	0.45
C30	102818	21	1.59	0.73
C60	85408	14	1.47	0.72
PA3	80123	80	2.76	0.91
PA7	64749	68	2.10	0.77
PA14	48361	70	2.53	0.87
PA30	77803	63	2.57	0.89
PA45	98955	31	0.73	0.43
PA60	89039	17	1.11	0.62
PA75	90091	21	0.73	0.42
PA90	80582	16	0.74	0.47
PA105	83397	11	0.13	0.04
PB3	37162	82	2.67	0.88
PB7	84481	92	2.44	0.84
PB14	60180	85	2.31	0.8
PB30	68849	97	2.75	0.9
PB45	107646	45	1.30	0.52
PB60	95428	20	0.97	0.54
PB75	105106	33	1.24	0.53
PB90	65624	17	0.48	0.27
PB105	86020	14	0.42	0.2
PC3	76095	83	2.53	0.87
PC7	72866	68	2.38	0.86
PC14	51027	88	2.51	0.85
PC30	78121	59	2.08	0.82
PC45	43784	60	2.50	0.88
PC60	90508	36	0.65	0.23
PC75	64176	55	2.39	0.87
PC90	64318	44	2.26	0.85
PC105	69903	25	0.85	0.47
PD3	49311	66	2.56	0.87
PD7	73380	85	2.54	0.88
PD14	57243	78	2.10	0.76
PD30	69923	62	2.54	0.88
PD45	74970	76	2.27	0.83
PD60	129557	14	0.25	0.09

PD75	87593	15	0.05	0.01
PD90	49819	32	1.24	0.48
PD105	76186	17	0.19	0.06
